# Reply to: ‘Flooding is a key driver of the Tonle Sap dai fishery in Cambodia’

**DOI:** 10.1038/s41598-021-81437-8

**Published:** 2021-02-15

**Authors:** Gaël Grenouillet, Kevin S. McCann, Bailey C. McMeans, Evan Fraser, Nam So, Zeb S. Hogan, Sovan Lek, Peng Bun Ngor

**Affiliations:** 1 CNRS, Université Toulouse III Paul Sabatier, IRD, UMR5174 EDB (Laboratoire Évolution and Diversité Biologique), 118 route de Narbonne, 31062 Toulouse, France; 2 Institut Universitaire de France, Paris, France; 3 University of Guelph, Guelph, ON Canada; 4 University of Toronto Mississauga, Mississauga, ON Canada; 5 Mekong River Commission Secretariat, Vientiane, Lao People’s Democratic Republic; 6 Global Water Center & Department of Biology, University of Nevada, 1664 N. Virginia Street, Reno, NV 89557 USA; 7 Wonders of the Mekong Project & Inland Fisheries Research and Development Institute, Fisheries Administration, No. 186, Preah Norodom Blvd., Khan Chamcar Morn, P.O. Box 582, Phnom Penh, Cambodia

**Keywords:** Food webs, Evolutionary developmental biology

**replying to**: A. S. Halls and K. G. Hortle; *Scientific Reports* 10.1038/s41598-021-81248-x (2021).

## Introduction

We empirically analyzed^1^ an industrial-scale ‘*Dai*’ fishery (2000/2001–2014/2015) presenting the signatures of indiscriminate fishing effects on the Tonle Sap’s fish community. Halls and Hortle^2^ suggest that apparent recent changes in Tonle Sap’s fish catch are more likely to reflect changing hydrological conditions than fishing-down effects, possibly caused by climate change and hydropower development. In addition, they question (1) the use of the *Dai* fishery data from 2000/01 onwards, as the fishery has been assessed since 1994^3,4^; (2) the *Dai* data being generated from a ‘standardized biological catch assessment’; (3) the explanation of compensatory response from small-bodied species in stabilizing the *Dai* seasonal catches; and (4) the mean fish weight used in^1^ being subject to ‘sampling related bias’. Finally, they claim that fishing effort from Tonle Sap may have declined as a result of fishing lot removal since 2012, and conclude that our findings may distract attention from irreversible and growing threats to fisheries caused by ongoing hydropower dam developments. We appreciate Halls and Hortle’s contributions and the opportunity to discuss these issues.

**Figure 1:**
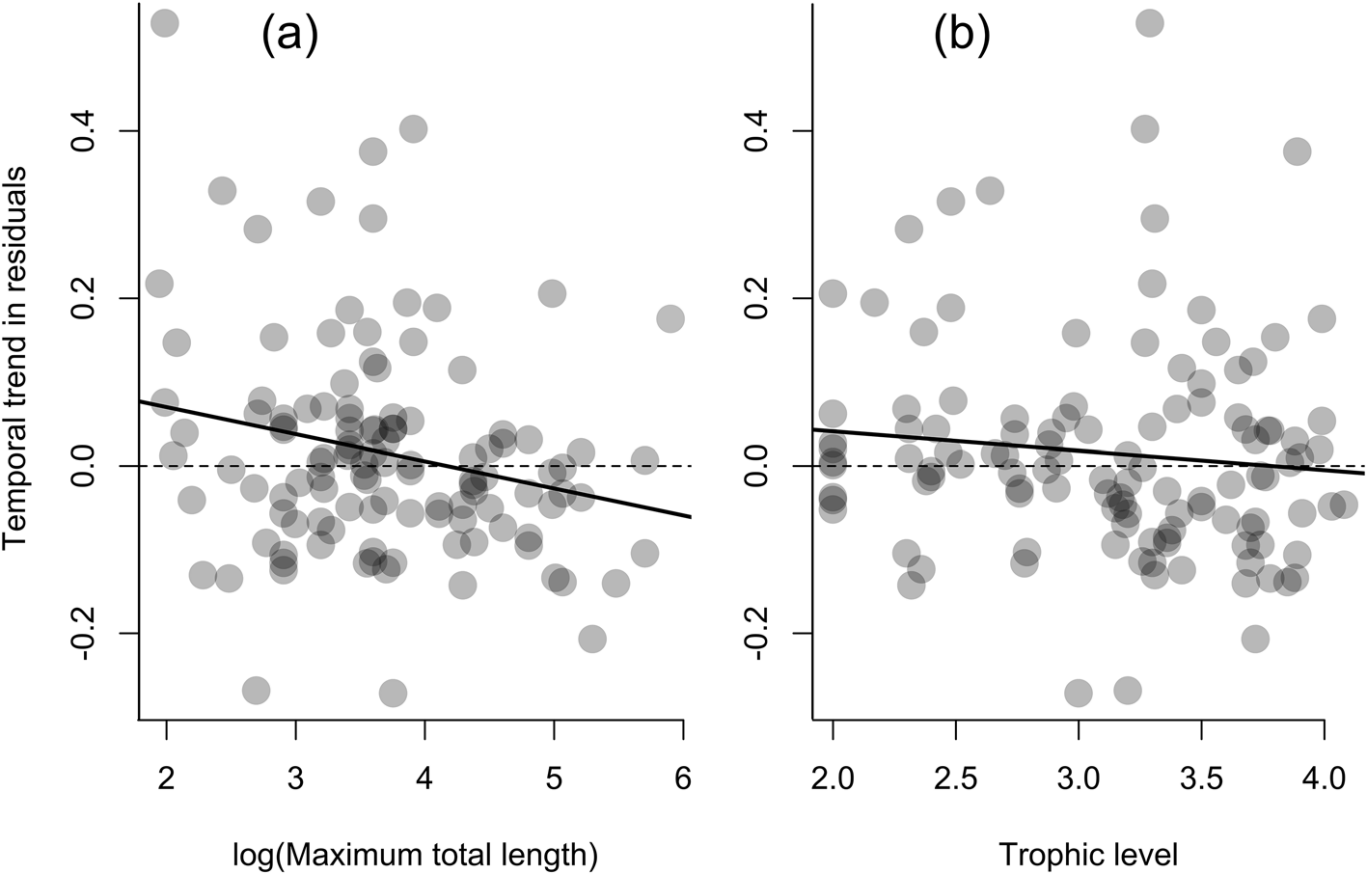
Temporal trend in residuals from the linear models relating species’ catch to Flood Index and water temperature against (**a**) log-transformed fish species’ maximum total length and (**b**) trophic level. The results indicated that 50% of species showed a declining trend in residuals, and this decline was significantly stronger for large-bodied fish species (p-value = 0.016).

To begin with, we re-analyzed our data and quantified temporal trends in species’ catch while accounting for both Flood Index (FI) suggested by Halls and Hortle and water temperature effects. To do this, we computed FI using mean daily water level data from Kampong Loung (Tonle Sap Lake) following^4^ and mean annual water temperature at Prek Kdam in Tonle Sap River where the *Dai* fishery operates. We then computed for each species a linear model expressing species abundance as a function of the two drivers, and assessed temporal trends in the residuals of these models (i.e. the part of variation not explained by FI and water temperature). The results indicated that 50% of species still showed a declining trend in residuals, with declines significantly more pronounced for large-bodied species (p-value = 0.016, Fig. 1). Here, we reiterate that our results support the findings of others, stressing the increasing contribution of catches of small-bodied species and the decline in the average fish size of the lower Mekong fisheries, both signs of overfishing^5–7^ and indiscriminate fishing effects^8,9^.

We analyzed the *Dai* data using 14 rows (*Dai* row 2–15) from 2000/01 onwards because of changes in sampling schemes and sampling intensity applied to assess the fishery. From 2000/01 onwards, *Dai* row 1 was discontinued, and it was only from that time that sampling intensity was relatively stable (see Halls et al*.* 2013, Table 11^4^) with *Dai* rows and their location unchanged. Although variation in the use of net types and mesh sizes may happen, the *Dai* gear is long-lasting of about 7 years on average^10^ and those gears are used seasonally to exploit fish systematically. This makes it seasonally comparable, particularly when the catch is assessed for the entire fishery.

By re-analysing the trends of seasonal catch of the three most prolific small species of the genus *Henichorynchus,* Halls and Hortle showed no compelling evidence of a compensatory response by small species, but did not consider the increasing trends of other small-bodied species (e.g. *Labiobarbus lineatus*) that were reported in the *Dai* catch (see Ngor et al.’s Supplementary Information Table S6^1^). Moreover, Halls and Hortle claim that fishing effort has declined, referring to the fisheries reform in 2012^11^. This reform has led to the establishment of 516 community fisheries (CFi) countrywide. While such policy changes open larger space for local community participation, Cambodia is suffering from governance challenges to successfully implement this policy. The challenges include unclear roles and responsibilities of stakeholders, poor coordination among resource management agencies, limited decentralization of roles and responsibilities to the CFis, weak capacity of the CFi to manage their local resources, insufficient funding to implement the policy and strong livelihoods dependency of the local communities on fisheries, finally resulting in intensification of fishing effort and conflicts over access rights to fisheries resources^12–17^. Fishers indeed borrow money to buy fishing gears, and felt compelled to catch as many fish as possible to repay their loans and meet the household needs^15^. There is no data on changes in fishing effort, so the claims made by Halls and Hortle on its decrease since 2012 cannot be empirically confirmed or denied. Our reading of the situation is that the fisheries remain under intense pressure despite attempts to implement improved fisheries governance.

Finally, while Tonle Sap remains one of the world’s largest inland fisheries, the system is facing many challenges, including intensive fishing pressure, with most large-sized Mekong fishes listed as threatened by IUCN and unsustainable fishing identified as a primary threat^18^. Our results do not imply that other impacts are unimportant, but rather that multiple (and potentially synergistic) stressors likely deteriorate the current status of the Tonle Sap fisheries^19,20^ and need to be further assessed and considered in the decision-making process.

## References

[1] Ngor PB (2018). Evidence of indiscriminate fishing effects in one of the world’s largest inland fisheries. Sci. Rep..

[2] Halls AS, Hortle KG (2021). Flooding is a key driver of the Tonle Sap dai fishery in Cambodia. Sci. Rep..

[3] Lieng S, Yim C, van Zalinge N (1995). Freshwater fisheries of Cambodia, I: The bagnet (Dai) fishery in the Tonle Sap River. Asian Fish. Sci..

[4] Halls, A. S. *et al. The Stationary Trawl (Dai) Fishery of the Tonle Sap-Great Lake System, Cambodia*. (Mekong River Commission, 2013).

[5] Hortle, K. G., Lieng, S. & Valbo-Jorgensen, J. *An introduction to Cambodia’s inland fisheries. Mekong Development Series No. 4* (Mekong River Commission, 2004).

[6] MRC. *State of the Basin Report 2010*. (Mekong River Commission, 2010).

[7] Allan JD (2005). Overfishing of Inland Waters. Bioscience.

[8] McCann KS (2016). Food webs and the sustainability of indiscriminate fisheries. Can. J. Fish. Aquat. Sci..

[9] KC, K. B. *et al.* Exploring tropical fisheries through fishers’ perceptions: Fishing down the food web in the Tonlé Sap, Cambodia. *Fish. Manag. Ecol.* **24**, 452–459 (2017).

[10] Hap, N. & Ngor, P. B. An economic analysis of fish production in the Dai Fisheries in Phnom Penh/Kandal Province, Cambodia. in *Cambodia fisheries technical paper series, Volume III* (eds. Zalinge, N. van, Nuov, S., Ounsted, R. & Lieng, S.) (The Management of the Freshwater Capture Fisheries of Cambodia Component of the Mekong River Commission’s Program for Fisheries Management and Development and the Department of Fisheries, 2001).

[11] Cooperman MS (2012). A watershed moment for the Mekong: newly announced community use and conservation areas for the Tonle Sap Lake may boost sustainability of the world’s largest inland fishery. Cambodian J. Nat. History.

[12] Thol D, Sato J (2015). The cost of privatizing the commons: Overlapping property systems in Tonle Sap Cambodia. Int. J. Commons.

[13] Thol D, Sato J (2014). Is greater fishery access better for the poor? Explaining de-territorialisation of the Tonle Sap Cambodia. J. Dev. Stud..

[14] Ratner BD (2006). Policy review community management by decree? Lessons from Cambodia’s fisheries reform. Soc. Nat. Resour..

[15] Ratner BD, So S, Mam K, Oeur I, Kim S (2017). Conflict and collective action in Tonle Sap fisheries: Adapting governance to support community livelihoods. Nat. Resour. Forum.

[16] Chap, S., Touch, P. & Diepart, J.-C. Fisheries reforms and right-based fisheries: Insights from community fisheries across Cambodia. (2016).

[17] Chan B, Ngor PB, Hogan ZS, So N, Brosse S (2020). Temporal dynamics of fish assemblages as a reflection of policy shift from fishing concession to co-management in one of the world’s largest tropical flood pulse fisheries. Water.

[18] Campbell T, Pin K, Ngor PB, Hogan Z (2020). Conserving Mekong Megafishes: Current status and critical threats in Cambodia. Water.

[19] Ngor, P. B., Lek, S., McCann, K. S. & Hogan, Z. S. Dams threaten world’s largest inland fishery. *Nature* **563**, (2018). 10.1038/d41586-018-07304-1 30405223

[20] Arias M, Holtgrieve G, Ngor PB, Dang TD, Piman T (2019). Maintaining perspective of ongoing environmental change in the Mekong floodplains. Curr. Opin. Environ. Sustain..

